# Multi-panel immunofluorescence analysis of tumor infiltrating lymphocytes in triple negative breast cancer: Evolution of tumor immune profiles and patient prognosis

**DOI:** 10.1371/journal.pone.0229955

**Published:** 2020-03-09

**Authors:** Ting-Fang He, Susan E. Yost, Paul H. Frankel, Andrew Dagis, Yu Cao, Roger Wang, Anthony Rosario, Travis Yiwey Tu, Shawn Solomon, Daniel Schmolze, Joanne Mortimer, Peter Lee, Yuan Yuan

**Affiliations:** 1 Department of Immuno-Oncology, City of Hope National Medical Center, Duarte, California, United States of America; 2 Department of Medical Oncology & Therapeutics Research, City of Hope National Medical Center, Duarte, California, United States of America; 3 Department of Biostatistics, City of Hope National Medical Center, Duarte, California, United States of America; 4 Department of Pathology, City of Hope National Medical Center, Duarte, California, United States of America; University of Tennessee Health Science Center, UNITED STATES

## Abstract

The evolutionary changes in immune profiles of triple negative breast cancer (TNBC) are not well understood, although it is known that immune checkpoint inhibitors have diminished activity in heavily pre-treated TNBC patients. This study was designed to characterize immune profile changes of longitudinal tumor specimens by studying immune subsets of tumor infiltrating lymphocytes (TILs) in paired primary and metastatic TNBC in a cohort of “poor outcome” (relapsed within 5 years) patients. Immune profiles of TNBCs in a cohort of “good outcome” (no relapse within 5 years) patients were also analyzed. Immune subsets were characterized for CD4, CD8, FOXP3, CD20, CD33, and PD1 using immuno-fluorescence staining in stroma, tumor, and combined stroma and tumor tissue. TIL subsets in “good outcome” versus “poor outcome” patients were also analyzed. Compared with primary, metastatic TNBCs had significantly lower TILs by hematoxylin and eosin (H&E) staining. Stromal TILs (sTILs), but not tumoral TILs (tTILs) had significantly reduced cytotoxic CD8^+^ T cells (CTLs), PD1^+^ CTLs, and total PD1^+^ TILs in metastatic compared with matched primary TNBCs. Higher PD1^+^ CTLs, PD1^+^CD4^+^ helper T cells (PD1^+^T_CONV_) and all PD1^+^ T cells in sTILs, tTILs and total stromal and tumor TILS (s+tTIL) were all associated with better prognosis. In summary, TIL subsets decrease significantly in metastatic TNBCs compared with matched primary. Higher PD1^+^ TILs are associated with better prognosis in early stage TNBCs. This finding supports the application of immune checkpoint inhibitors early in the treatment of TNBCs.

## Introduction

Triple negative breast cancer (TNBC) is an aggressive form of breast cancer (BC) characterized by poor overall survival upon diagnosis of metastases. Unlike hormone receptor positive tumors and HER2/neu positive tumors, TNBCs lack effective targeted therapies. Among all BCs, TNBCs are the most immunogenic due to the relatively high level of tumor infiltrating lymphocytes (TILs)[1–3]. Increased stromal TILs are associated with significantly improved response to neoadjuvant chemotherapy and favorable long term survival in breast cancer[4–6].

Currently, immune checkpoint inhibitors have gained success in multiple solid tumors such as melanoma, lung cancer and renal cell carcinoma, and checkpoint inhibitors are now undergoing rigorous investigation in clinical trials for treatment of TNBC[7–9]. The efficacy of immune checkpoint inhibitors appears to vary significantly among patients with variable clinical setting. In patients with heavily pre-treated metastatic disease, the single agent checkpoint inhibitor pembrolizumab has a much lower response rate of 5% compared with 19%-23% in patients who received limited lines of therapy[8–10]. In neoadjuvant trials, adding pembrolizumab to conventional chemotherapy increased pathological complete response (pCR) from approximately 20% to 62% (n = 21) in ISPY-2[7]. In addition, the overall response rate of atezolizumab was empirically higher in first-line than in second-line or greater patients (24% vs. 6%)[11]. The variable efficacy data and the underlying mechanism of immune checkpoint inhibitors is not well understood.

TILs have been well-recognized as a prognostic biomarker in breast cancer. Despite the knowledge of the prognostic and predictive value of TILs, there is limited knowledge regarding the change in TILs and TIL subsets during metastasis and tumor recurrence. Since immune response is influenced by tumor progression and chemotherapy treatment selection, immune cell subset profile changes in the tumors and stroma need to be thoroughly studied in order to guide immunotherapy strategies. TIL quantification is currently performed using H&E staining according to the International Immuno-Oncology Biomarker Working Group on Breast Cancer guidelines[12]. Stromal TILs (sTILs) are located in the fibrous stroma adjacent to the tumor cells, and tumoral TILs (tTILs) have cell-to-cell contact with carcinoma cells. Assessing TILs in H&E stained slides remains the current gold-standard for assessing tumor immunogenicity[2, 13]. With accumulating evidence of the role of immune cell complexity in the tumor microenvironment, there is a critical need to understand the subsets and functionality of sTILs and tTILs in longitudinal primary and metastatic samples. In addition, there is a knowledge gap in understanding TILs and TIL subsets in “good outcome” (no relapse within 5 years) versus “poor outcome” TNBCs (relapse within 5 years).

The primary goal of this study is to understand the immune profiles of primary and metastatic TNBC to capture the changes in total TILs and TIL subsets in the “poor outcome” patients. We also evaluated the association of these TIL subsets with clinical outcome in a larger cohort of patients, including a cohort of “good outcome” patients.

## Materials and methods

### Patient selection

TNBC specimens were selected as part of a COH IRB-approved retrospective protocol via the COH Biorepository from patients diagnosed and treated at COH from 2002 to 2012. Estrogen receptor (ER), progesterone receptor (PR), and HER2/neu testing was performed on both primary and metastatic tumors. Studies involving human participants were aligned with the ethical standards of COH, the national research committee, and the Helsinki Declaration of 1964 and amendments. All participants provided written informed consent. Eligible patients had the following features: TNBC defined by ASCO/CAP guidelines; at least one tumor biospecimen available from initial surgery or biopsy; and clinical outcome data available for identification of relapse free survival. All samples were formalin-fixed paraffin-embedded (FFPE) tissue. Chart review was performed by two independent investigators to collect patient characteristics and treatment variables. Demographic data including age, date of birth, date of diagnosis, date of relapse, and date of death (if applicable) was obtained. Tumor characteristics such as receptor status, tumor size, grade, stage, and breast cancer type were obtained, as well as treatment variables such as chemotherapy and radiation. Two cohorts of patients were studied: “poor outcome TNBC” (n = 17) defined by recurrence of disease within 5 years of initial surgery, of which 2 were excluded from outcomes due to met-met biopsy samples only, and “good outcome TNBC” (n = 16) defined by no recurrence of disease within 5 years of initial surgery. All 33 patients had at least one primary tumor tissue available for multi-color immunofluorescence analysis of TIL subsets. A total of 10 patients of the 17 “poor outcome” group had paired primary-metastatic specimens, 2 had paired metastatic-metastatic specimens, and 5 patients had only primary tumor for TIL subset comparison. Out of all 33 patients, 5/45 specimens (11%) were not included in the final analysis due to poor tumor/stromal separation (N = 4) and insufficient tissue (N = 1).

### Histological assessment

All histopathological parameters used in this study were directly documented from the original pathology reports after being reviewed by two independent pathologists. Immunohistochemistry (IHC) staining was performed with an automated staining module with supplied antibodies against ER, PR, and HER2 according to manufacturer’s instruction manual (BenchMark ULTRA IHC/ISH Staining Module, Ventana Medical Systems, Tucson, AZ, USA). ER, PR, and HER2 status was determined on formalin-fixed paraffin-embedded (FFPE) biopsies using ASCO/CAP guideline. TIL quantification was performed using the International TILs Working Group 2014 Guideline [12].

### Immunofluorescence and multispectral imaging analysis

Immunofluorescence analysis of TIL and TIL subsets were performed with methods described previously[14]. Commercial Vectra^®^ 3.0 Automated Quantitative Pathology Imaging System (Akoya Biosciences) was used to acquire images and inForm^®^ Image Analysis Software (Akoya Biosciences) was used to perform image analysis. With InForm^®^ software, a machine-learning approach is used for phenotyping to identify and classify cells. In each sample, the images from the whole slide were analyzed, and the MSI of more than 50% of the slide was reported, with the exception of excluded regions that had artificial effect or poor quality. Depending on the size of the sample, the number of MSIs varied, and an average cell frequency was used in this analysis. The antibodies were: anti-human CD4 antibody (Dako, 4B12); anti-human CD8 antibody (Biocare, SP16); anti-human CD20 antibody (Biocare Medical, L26); anti-human PD1antibody (Origene, UMAB199); anti-human FOXP3 antibody (Biocare, 236A/E7); and anti-human CD33 antibody (Leica, PWS44).

### Clinicopathological analysis

Patient characteristics were captured including age, receptor status, tumor size, grade, stage, breast cancer type, chemotherapy and radiation history, recurrence free survival (RFS), and overall survival (OS). RFS was defined as date of surgery to date of first relapse, and OS was defined as date of surgery to date of death.

### Statistical plan

The fold change of TIL subsets in paired early versus later biopsy TNBCs (N = 10 prim-met TNBCs; N = 2 met-met TNBCs) are plotted on a log plot (log_2_) using the ratio of later versus early specimens. Medians of the log are indicated to show increase or decrease in the transformed TILs count and median fold change and interquartile range are presented. Wilcoxon signed-rank tests were performed to test whether the changes were significantly different from 1 (i.e. no change). Up-arrow ▲ indicates undetectable baseline TILs, and down-arrow ▼ indicates undetectable second time point TILs. ■ indicates that a metastatic event was the base-line, with another metastatic event as the second time point. If one measurement (single dagger) or both measurements (double dagger) are missing due to technical difficulty of staining or insufficient tissue, then no fold-change was reported.

Box and whisker plots were used to show baseline TILs. Patients are separated by “good outcome” (n = 16, no relapse within 5 years) and “poor outcome” (n = 17, relapse within 5 years). Raw data values recorded as being “below the limit of detection” were assigned values of 0.01, which is less than the value of any positive measurement. This was done to include negative results in the analyses and distinguish them from missing data. Only correlations significant when adjusted for multiple comparisons were reported.

Patient groups were chosen by survival outcome, the “good outcome” group having been defined as patients with no cancer recurrence within 5 years after initial surgery. The “poor outcome” group was sampled as all available specimens from patients who had recurrence within 5 years of initial curative-intent surgery.

## Results

### Patient characteristics and treatment history

**Fig 1:**
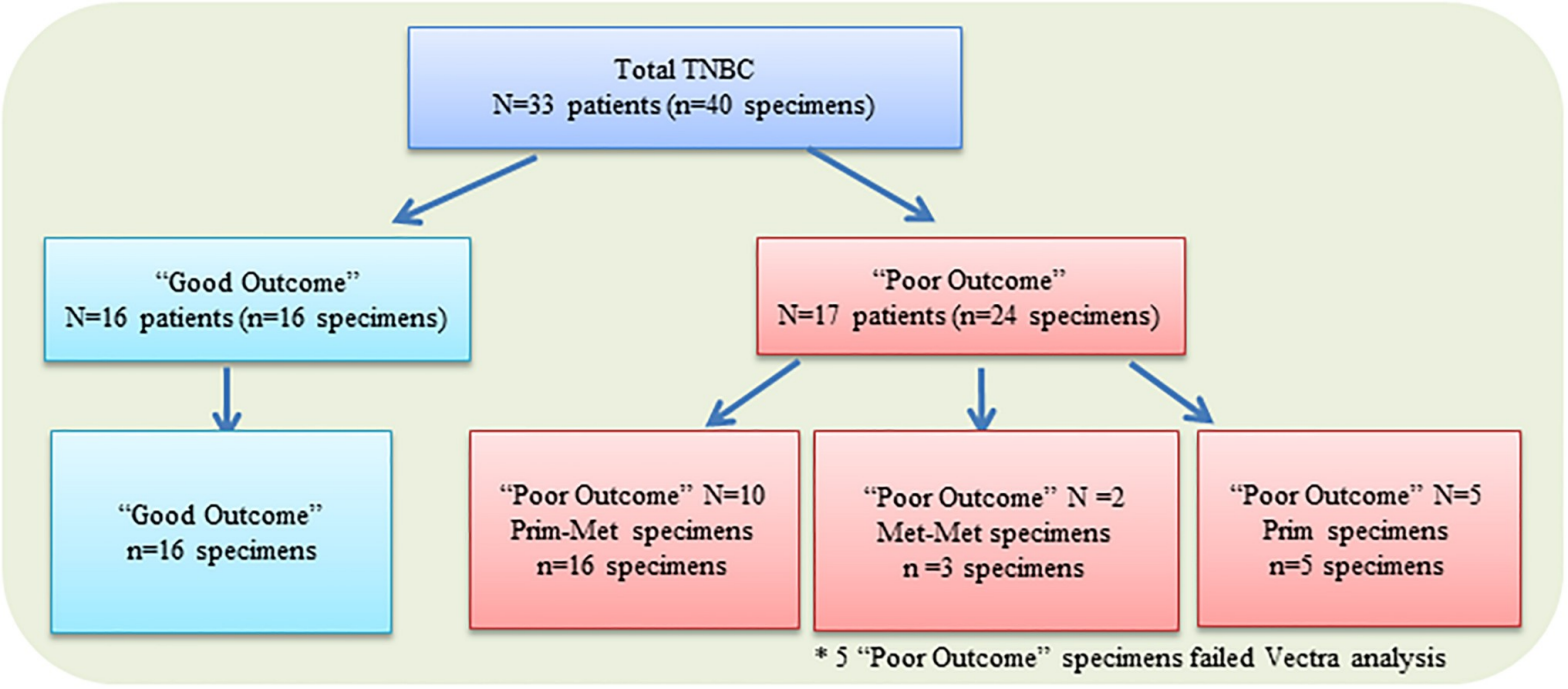
Diagram of patients and samples. Out of 33 patients, there were 16 “good outcome” patients and 17 “poor outcome” patients included in this study. Of the 17 “poor outcome” patients, 10 had both primary and metastatic paired tissue (N = 10 pts, n = 20 specs), 2 had paired metastatic samples (N = 2 pts, n = 4 specs), and 5 had only primary tumor (N = 5 pts, n = 5 specs) for immunofluorescence analysis. Five specimens failed Vectra analysis due to technical difficulties (4 failed due to lack of stroma/tumor separation and 1 failed due to insufficient tissue for analysis). N = number of patients; n = number of specimens; Pts, Patients; Specs, specimens.

A summary of patients and samples is shown in Fig 1. There were 16 “good outcome” patients, and 17 “poor outcome” patients, including 10 patients with both primary and metastatic biopsy samples, 2 patients with paired metastatic samples (primary sample were not available; metastatic samples were longitudinal in time), and 5 patients with only primary tumor available for further analysis. The clinical characteristics, pathological features, and treatment histories of the 33-patient cohort are described in Table 1. The clinicopathological features of the “good outcome” and “poor outcome” groups are similar with the exception of (neo) adjuvant chemotherapy and radiation history, where a lower percentage of “poor outcome” patients received anthracycline-containing chemotherapy and radiation. Median relapse-free survival (RFS) for the “poor outcome” TNBCs was 16 months (range 1 to 39 months). “Poor outcome” patients had a median overall survival (OS) of 38.5 months (range 8–119). By definition, none of the “good outcome” TNBCs relapsed within 5 years, with a median follow-up of 115.5 months (range 66–158).

**Table 1:** Patient characteristics and treatment history (N = 33, all TNBCs^†^).

	Poor Outcome	Good Outcome
	(N = 17 pts)	(N = 16)
Median age (range)	51 (40–82)	55.5 (27–76)
Primary tumor size (cm), median (range)	2.5 (1.4–5.0)	3.1 (1.3–4.7)
Tumor grade, N (%)		
1	0 (0%)	0 (0%)
2	4 (24%)	2 (12.5%)
3	13 (76%)	14 (87.5%)
Primary cancer stage, N (%)		
I	4 (23%)	3 (18.8%)
II	11 (65%)	10 (62.4%)
III	2 (12%)	3 (18.8%)
Breast Cancer Type		
IDC	14 (82%)	16 (100%)
Other	3 (18%)	0 (0%)
(Neo) adjuvant chemotherapy		
Anthracycline-containing	10 (59%)	14 (87.4%)
Non-anthracycline-containing	2 (12%)	0 (0%)
None	4 (23%)	1 (6.3%)
Unknown	1 (6%)	1 (6.3%)
Radiation		
Yes	6 (35%)	10 (62.5%)
No	5 (30%)	1 (6.3%)
Unknown	6 (35%)	5 (31.2%)
Sites of metastasis		
Lymph nodes	4	N/A
Lung	2	N/A
Bone	3	N/A
Brain	3	N/A
Other	6	N/A

^†^Receptor status: all TNBC with the exception of one primary tumor that had a phenotype shift from ER^+^ to TNBC

### Stromal TILs quantification by H&E staining in metastatic TNBC compared with paired primary

TILs quantification was performed on paired TNBCs (10 prim-met pairs, 2 met-met pairs) with H&E staining using the International Immuno-Oncology Working Group Guideline[12]. Of the 10 primary tumors, 3 had high (≥ 50%) sTILs and 7 had low (≤20%) TILs. (Table 2). Out of the 10 paired prim-met specimens, sTILs decreased in 4 patients, were unchanged in 3 patients, and increased slightly in 3 patients.

**Table 2:** Stromal TIL quantification by H&E (n = 12).

Patient	Tissue Type	H&E sTILs (%)	LPBC
1.1	Breast prim	60	Yes
1.2	Lung met	60	Yes
2.1	Breast prim	60	Yes
2.2	Bone met	30	No
3.1	Breast prim	70	Yes
3.2	LN met	50	Yes
4.1	Breast prim	10	No
4.2	Chest wall met	5	No
5.1	Brain met	10	No
5.2	Soft tissue met	3	No
6.1	Breast prim	20	No
6.2	LN met	40	No
7.1	Endometrium met	5	No
7.2	LN met	2	No
8.1	Right breast prim	5	No
8.2	Left breast met	10	No
9.1	Breast prim	10	No
9.2	Brain met	10	No
10.1	Breast prim	5	No
10.2	Skin met	1	No
11.1	Breast prim	10	No
11.2	Bone met	30	No
12.1	Breast prim	10	No
12.2	Lung met	10	No

TIL, tumor infiltrating lymphocytes; LPBC, Lymphocyte-predominant breast cancer [15] (defined as ≥50% stromal TILs)

### Stromal CTL, PD1^+^ CTL, and total PD1^+^ subsets are significantly reduced as the disease advances in TNBCs

**Fig 2:**
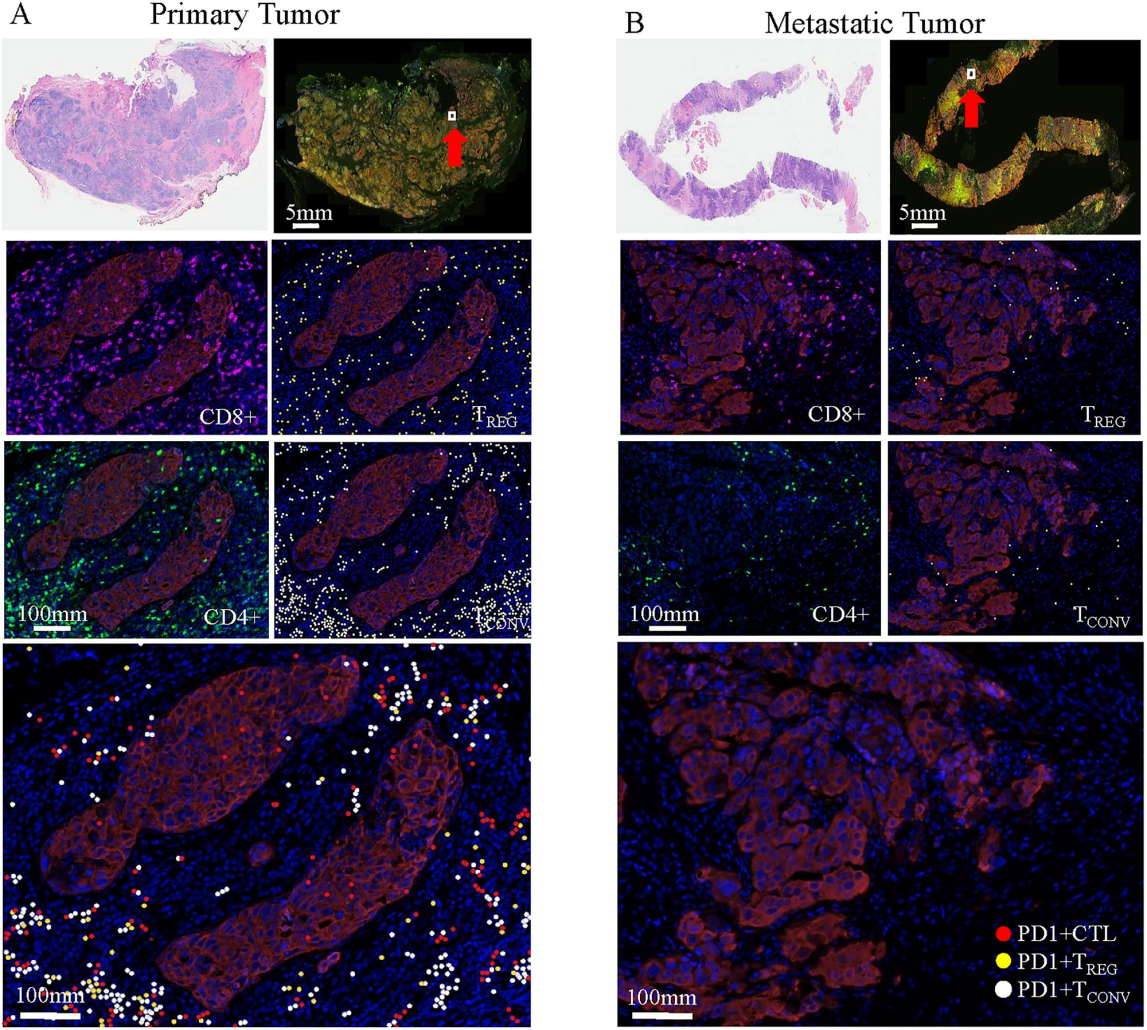
Representative immunofluorescence staining of TILs in primary and metastatic tumors. CD8^+^, CD4^+^, T_REG_ (CD4^+^ FOXP3^+^), T_CONV_ (CD4^+^ FOXP3^-^), PD1^+^ CTL (CD8^+^ PD1^+^), PD1^+^ T_REG_, and PD1^+^ T_CONV_. VECTRA analysis revealed higher TILs in primary tumor (A) than metastatic tumor (B). Red arrow, area of VECTRA analysis; scale bar 5 mm for H&E and IF; scale bar 100 μm for all other panels.

**Fig 3:**
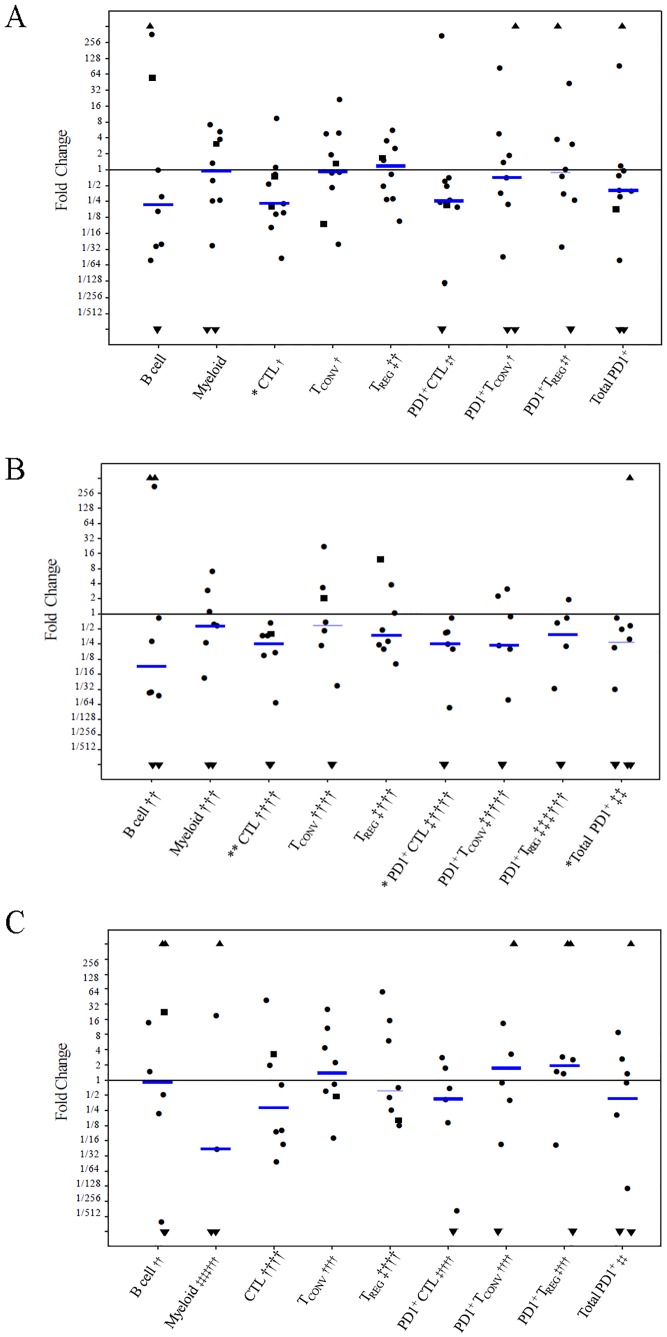
Fold change of TIL subsets in paired TNBCs (N = 12 pts including 10 prim-met, and 2 met-met specimens) using log2 ratio of metastatic versus primary TNBC (n = 10, ●) or second recurrence versus first recurrence (n = 2, ■). A) Fold change of s+tTILs in combined stromal and tumor tissue; B) Fold change of sTILs in stromal tissue; C) Fold change of tTILs in tumor tissue. ● prim-met specimens; ■ met-met specimens; ▲ undetectable baseline TILs; ▼ undetectable 2^nd^ time point TILs; ‡ both measurements are zero; † at least one measurement is missing value(s); B cells (CD20^+^), myeloid cells (CD33^+^), CTLs (CD8^+^), T_CONV_ (CD4^+^ FOXP3), T_REG_ (CD4^+^ FOXP3^+^), PD1^+^ CTL (PD1^+^ CD8^+^), PD1^+^ T_CONV_ (PD1^+^ CD4^+^ FOXP3^-^), PD1^+^ T_REG_ (PD1^+^ CD4^+^ FOXP3^+^), and total PD1^+^ cells (PD1^+^ CD8^+^/ PD1^+^ T_CONV_ / PD1^+^ T_REG_); *p ≤ 0.05; ** *p* ≤ 0.01.

Immunofluorescence staining using multiplex antibodies for TIL subsets was performed. Representative stains of cell surface markers in primary and metastatic tumors are shown in Fig 2. Precise identification of the tumor area and enumeration of immune cell subtypes in sTILs and tTILs were performed. Fold changes of TIL subsets, expressed as the ratio of metastatic versus primary TNBC (n = 10) combined with second metastases versus first metastases (n = 2), were analyzed (Fig 3). Baseline TILs were not detectable in 1 primary and 4 metastatic samples by VECTRA analysis. Fold changes greater than 1 represented an increase of TILs from primary to metastatic tumor (or first metastases to second), and values less than 1 represented a decrease of TILs from primary to metastatic tumor. The fold changes were plotted on a log_2_ scale. The following groups of cells were analyzed for total TILs (s+tTILs), stromal TILS (sTILs), and tumor TILs (tTILs) as shown in Fig 3. B cells (CD20^+^), myeloid cells (CD33^+^), CTLs (CD8^+^), T_CONV_ (CD4^+^ FOXP3^-^), T_REG_ (CD4^+^ FOXP3^+^), PD1^+^ CTL (PD1^+^ CD8^+^), PD1^+^ T_CONV_ (PD1^+^ CD4^+^ FOXP3^-^), PD1^+^ T_REG_ (PD1^+^ CD4^+^ FOXP3^+^) were assayed, and we also reported PD1^+^ TILs as the sum of the three PD1^+^ subsets. Total s+tCTLs were reduced to about 1/4 (median fold change 0.23 [IQR 0.15, 0.78], p = 0.04, Wilcoxon signed-rank test) comparing second specimen (metastasis) vs. first specimen (primary or metastasis) (Fig 3A). Immune subsets showed a consistent pattern of decreased number in second samples for sTILs (Fig 3B), with significant findings for CTLs (approximately 1/4 of the baseline value, with a median fold change of 0.25 [IQR 0.085, 0.388], p = 0.008), as well as for PD1^+^ CTLs (approximately 1/4 of the baseline value, with a median fold change of 0.25 (interquartile range, [IQR, 0.05, 0.44], p = 0.02), and total PD1^+^ cells (approximately 1/3 of the baseline value, median fold change of 0.3 [IQR <0.01, 0.6], p = 0.04). Tumor TILs were more variable (Fig 3C), and no subsets were statistically significant by Wilcoxon signed-rank test.

### Total s+tTILs and TIL subsets are associated with outcome

**Fig 4:**
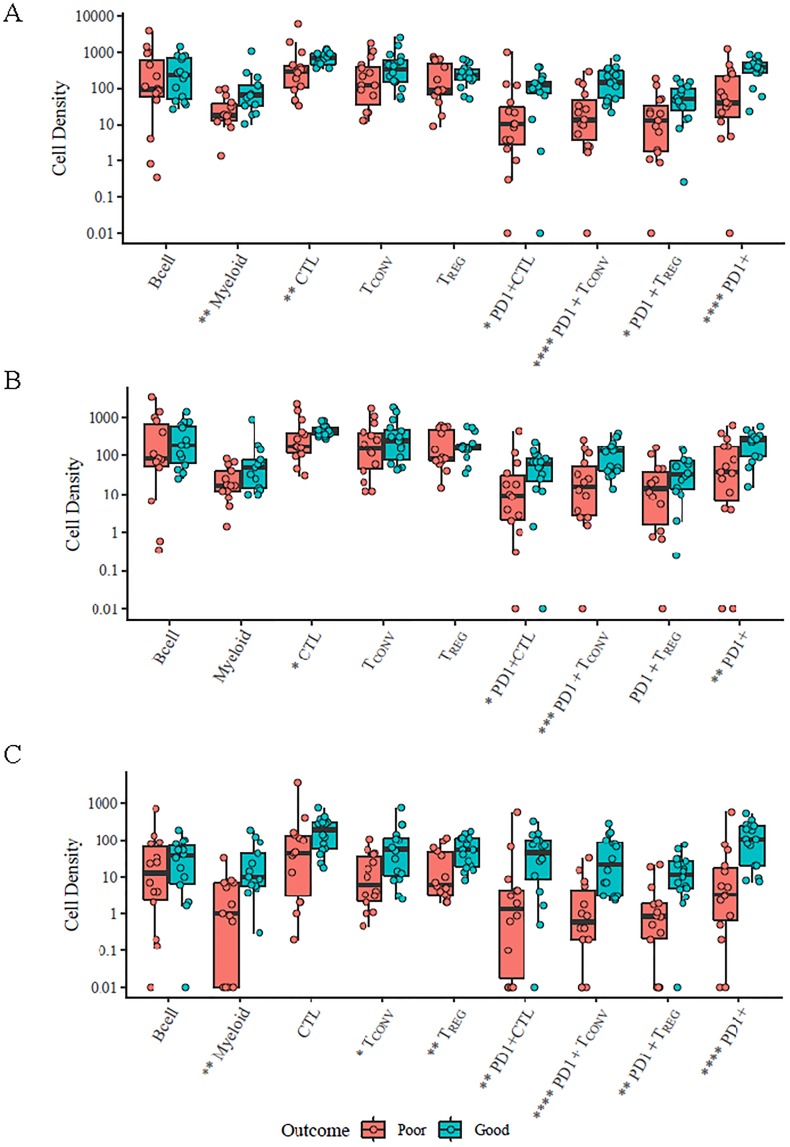
Association of TILs in primary TNBC with 16 “good outcome” (no relapse within 5 years) versus 15 “poor outcome” (relapsed within 5 years; 2 patients did not have primary specimen). A) Association of combined s+tTILs in “good vs. poor outcome” TNBC; B) Association of sTILs in “good vs. poor outcome” TNBC; and C) Association of tTILs in “good vs. poor outcome” TNBC. Red, poor outcome; blue/green, good outcome; *p ≤ 0.05; **p ≤ 0.01; ***p ≤ 0.005; ****p ≤ 0.001.

TIL subsets from two cohorts of TNBCs with distinctive clinical outcomes were compared (Fig 4). The “good outcome” cohort was defined as patients with no cancer recurrence within 5 years after initial surgery. The “poor outcome” group was defined as patients who had recurrence within 5 years of initial curative-intent surgery. Compared with patients with “good outcome” tumors, patients in the “poor outcome” group had a statistically significant decrease in TILs.

In combined stromal and tumoral TILs, median s+tTIL subset densities (cells/mm^2^) differentiating “poor outcome” versus “good outcome” were as follows (Table 3): myeloid (p = 0.008); CTL (p = 0.007); PD1^+^ CTL (p = 0.02); PD1^+^ T_CONV_ (p = 0.0009); PD1^+^ T_REG_ (p = 0.03); and all PD1^+^ cells (p = 0.001). In stromal TILs, median sTILs subsets differentiating “poor outcome” vs. “good outcome” were as follows: CTL (p = 0.03); PD1^+^ CTL (p = 0.05); PD1^+^ T_CONV_ (p = 0.004); and all PD1^+^ cells (p = 0.01). In tumoral TILs, median tTILs subsets differentiating “poor outcome” vs. “good outcome” were as follows: myeloid (p = 0.006); T_CONV_ (p = 0.02); T_REG_ (p = 0.009); PD1^+^ CTL (p = 0.009); PD1^+^ T_CONV_ (p = 0.0006); PD1^+^ T_REG_ (p = 0.002); and all PD1^+^ cells (p = 0.0006). Analyses were performed as two-sided Wilcoxon rank-sum test. In the tumor, increased T cell subsets, including myeloid cells, T_CONV_, T_REG_ and all PD1^+^ T cell subsets were associated with improved clinical outcome. In the stroma, increased CTL, PD1^+^CTL, PD1^+^T_CONV_, and total PD1^+^ T cells were associated with improved clinical outcome.

**Table 3:** Stromal and tumor TIL subset densities (cells/mm^2^) in good versus poor outcome patients.

	Poor outcome Median density (cells/mm^2^)	Good outcome Median density (cells/mm^2^)	p-value
Total stromal and tumor TILs (s+tTILs)			
Myeloid cells	17.9	66.04	0.008
CTL	298	693	0.007
PD1^+^ CTL	10.3	120.5	0.02
PD1^+^ T_CONV_	13.4	148.2	0.0009
PD1^+^ T_REG_	12.7	51.5	0.03
All PD1^+^ cells	40.3	393.6	0.001
Stromal TILs (sTILs)			
CTL	178.5	453	0.03
PD1^+^ CTL	9.2	61.5	0.05
PD1^+^ T_CONV_	16.5	131.5	0.004
All PD1^+^ cells	37.2	263.5	0.01
Tumor TILs (tTILs)			
Myeloid cells	1.0	10	0.006
T_CONV_	7.0	56.5	0.02
T_REG_	6.4	53.5	0.009
PD1^+^ CTL	1.4	41.5	0.009
PD1^+^ T_CONV_	0.65	23.0	0.0006
PD1^+^ T_REG_	0.85	12.0	0.002
All PD1^+^ cells	3.3	109.9	0.0006

Correlation analysis was performed in order to understand the relationship between TIL subsets and showed that PD1^+^ CTLs correlated with total PD1^+^ cells in both tTILs (rho = 0.99, p < 0.01) and sTILs (rho = 0.98, p < 0.01). In addition, PD1^+^ T_REG_ correlated with total PD1^+^ cells in tTILs (rho = 0.93, p = 0.03), but not sTILs (S2 Table).

## Discussion

Immune checkpoints inhibitors such as PD1 or PD-L1 inhibiting antibodies have shown promising activity in a subgroup of metastatic TNBC and newly diagnosed early stage TNBCs[7–9, 16, 17]. Multiple clinical trials testing efficacy of immune checkpoint inhibitors in combination with targeted therapies or chemotherapy in metastatic and early stage TNBCs are underway[18–22]. In KEYNOTE-86 trial, pembrolizumab (anti-PD1 antibody) showed a response rate of 5% in Cohort A (previously treated metastatic TNBC)[9] in contrast with a response rate of 23% in cohort B (previously untreated metastatic TNBC)[10]. In early stage TNBCs, there was a complete pathological response (pCR) rate of 62% in the neoadjuvant ISPY-2 trial with pembrolizumab added to chemotherapy) [7], a pCR rate of 50% with pembrolizumab plus nab-paclitaxel followed by pembrolizumab plus doxorubicin and cyclophosphamide, and a pCR rate of 80% in KEYNOTE-173 with pembrolizumab plus carboplatin and nab-paclitaxel followed by pembrolizumab plus doxorubicin and cyclophosphamide. The underlying mechanism of such a dramatic reduction in the response rate from neoadjuvant to the metastatic setting remains poorly understood. In the current study, we identified significantly reduced levels of stromal CTL and stromal PD1^+^ CTL in the metastatic TNBCs compared with matched primary TNBC. Subsequent metastatic disease had less TILs compared to first metastasis. This finding is consistent with the clinical trial observation and supports using immune checkpoint inhibitors earlier in the treatment of TNBC, which usually carries a very poor outcome once disease recurs.

Stromal TILs are a good prognostic factor and predictive of response to immune checkpoint inhibitors in breast cancer. Robust levels of stromal TILs have been associated with improved response to neoadjuvant therapy with increased pathological complete responses (pCR) in TNBC[5]. Higher TILs is also associated with improved disease-free and overall survival rates in TNBC[13, 23–25]. Besides TILs, increased programmed cell death protein 1 ligand (PD-L1)[26] expression on the surface of tumor, and increased tumor mutation burden represent potential predictive markers for immune checkpoint inhibitor response. Despite multiple studies focusing on total TIL counts, few have provided further insight on immune subsets. Cancer cells grow in a complex microenvironment composed of stromal cells, lymphoid cells, myeloid cells, cytokines, chemokines, vascular and lymphatic vessels. Analysis of TILs subsets is critical to understand the interaction of tumors with immune cells and stroma. In the current retrospective analysis of TIL subsets comparing stage-matched good versus poor prognostic patients receiving conventional chemotherapy and standard of care treatment, higher PD1^+^ CTLs, PD1^+^ T_CONV_ and all PD1^+^ T cells in sTILs, tTILs and s+tTILs were all associated with better prognosis. This study is novel in identifying subsets of TILs and their association with prognosis and survival in TNBCs.

The current study identified PD1^+^ T cells as a potential prognostic biomarker in TNBCs; however, the underlying mechanism remains poorly understood. PD1 is expressed on activated T cells, B cells, NK cells, macrophages and dendritic cells[27, 28]. PD1 receptor binding is involved in inhibition of activated T cells [27], but PD1 expression does not distinguish exhausted T cells from activated T cells. Further in-depth analysis is required to fully understand the critical role of PD1 in T cell function. Increased tumor CD33^+^ myeloid cells were associated with better prognosis in the current study. Previous evidence has shown CD33^+^ myeloid cells may be composed of both tumor-resident myeloid-derived suppressor cells (MDSCs) and activated neutrophils[29]. MDSCs are known to be associated with poor prognosis[29]. Future analysis is necessary to identify the role of each subpopulation of immune cells and their association with clinical outcome.

Despite accumulating data showing that TILs decrease after chemotherapy, the underlying mechanism of significantly reduced TILs and TIL subsets in post-neoadjuvant and metastatic TNBCs remains poorly understood. Dieci *et al*. reported that the presence of TILs in post-neoadjuvant residual tumor is associated with better outcome in TNBC patients[30]. This may indicate the significant impact of tumor microenvironment in the tumor-stroma-immune cell interaction. In more heavily pretreated TNBCs, cancer associated fibroblasts (CAFs) may limit T cell infiltration and dampen immune responses. This has been observed in human pancreatic cancer, where high levels of fibrosis are associated with poor CD8^+^ T cell infiltration[31]. The EMT phenotype in human breast cancer cells was associated with distinct morphologic changes and inhibition of TIL-mediated tumor cell lysis, which reduced the susceptibility of cancer cells to T cell–mediated immune surveillance[32, 33]. This may explain the reduced TILs and TIL subsets observed in tumors treated with multiple chemotherapies. Immune profiling of breast cancer stromal cells, TILs, and breast tumor cells can add substantially to breast cancer outcome and add to our understanding of the mechanism of relapse.

Our current study showed significantly decreased TILs and TIL subsets in metastatic TNBC compared with matched primary TNBC. This finding supports the more robust response to immune checkpoint inhibitors seen in early stage TNBCs compared to heavily pretreated metastatic TNBCs from recent clinical trials. Our current study is limited by small sample size, retrospective nature, and lack of immune therapy intervention. Our highly prognostic TILs and TIL subsets finding may be associated with response to immune checkpoint inhibitors. These findings will be further verified in ongoing clinical trials incorporating immune checkpoint inhibitors for treatment of metastatic breast cancer[18, 34].

## Conclusion

In this study, TILs and TIL subsets decreased significantly as disease advanced in TNBC. This confirms the more robust response to immune checkpoint inhibitors seen in early stage TNBCs compared to heavily pretreated metastatic TNBCs from recent clinical trials. TILs and TIL subsets carry important prognostic value in TNBCs. Higher PD1^+^ T cells are associated with better clinical outcome. These findings will be further verified in current clinical trials incorporating immune checkpoint inhibitors for treatment of metastatic TNBC.

## Supporting information

S1 Table Multispectral imaging and quantitative analysis. (XLSX)

S2 Table Correlation analysis of stromal and tumor T cell subsets with total T cells. (DOCX)
