# Supplementary material for: Multi-panel immunofluorescence analysis of tumor infiltrating lymphocytes in triple negative breast cancer: Evolution of tumor immune profiles and patient prognosis
Source: PLoS One. 2020 Mar 9;15(3):e0229955. doi: 10.1371/journal.pone.0229955 (PMC7062237; doi:10.1371/journal.pone.0229955)
Supplement: S2 Table — (DOCX) [file pone.0229955.s002.docx]

**S2 Table.** Correlation analysis

| Total = Stroma + Tumor, log base 2 transformation of (metastasis or second measurement / primary) | | | | |
| --- | --- | --- | --- | --- |
| Subset | Subset | N | Rho | P-value adjusted for multiple comparisons |
| PD1+ CTL | PD1+ | 10 | 0.98 | <0.01 |
| PD1+ CTL | PD1+ Treg | 9 | 0.91 | 0.03 |
| PD1+ | PD1+ Treg | 10 | 0.98 | <0.01 |
| Stroma, log base 2 transformation of (metastasis or second measurement / primary) | | | | |
| Subset | Subset | N | Rho | P-value adjusted for multiple comparisons |
| PD1+ CTL | PD1+ | 7 | 0.98 | <0.01 |
| Tumor, log base 2 transformation of (metastasis or second measurement / primary) | | | | |
| Subset | Subset | N | Rho | P-value adjusted for multiple comparisons |
| PD1+ CTL | PD1+ | 7 | 0.99 | <0.01 |
| PD1+ | PD1+ Treg | 8 | 0.93 | 0.03 |
